# 

**Published:** 1891-08-08

**Authors:** 


					renrenray-
AND INVALIDS.
for INFANTS
JRttlCH HOSPITAL
.saotv Western InfirmAa*
WITH EXTBA'.'.NURSINB supplement.
^^^C|No.254.Yol.X.] Satxtrday,
August 8 th., 1891.
[One Penny.

				

